# Inertia and Rapid Divergence in the Evolution of Yawning: A Comparison Between Two Closely Related but Socially Different Monkeys

**DOI:** 10.1002/ajp.70049

**Published:** 2025-05-29

**Authors:** Luca Pedruzzi, Veronica Maglieri, Paolo Oliveri, Martina Francesconi, Rea Riccobono, Filippo Bigozzi, Alban Lemasson, Elisabetta Palagi

**Affiliations:** ^1^ EthoS (Ethologie Animale et Humaine) U.M.R 6552, Université de Rennes, Université de Normandie, CNRS Rennes France; ^2^ Unit of Ethology, Department of Biology University of Pisa Pisa Italy; ^3^ Institut Universitaire de France France; ^4^ Natural History Museum University of Pisa Pisa Italy

**Keywords:** behavioral contagion, communication, contagious yawning, geladas, hamadryas baboons

## Abstract

Complex communication systems appear to evolve alongside social complexity. Comparing closely related species with similar social structures but distinct sociobiology offers valuable insights into the evolution of communicative variability. Here, we explore yawning (morphology, sensory modalities, contexts, contagious effect), a highly conserved behavioral trait, in two zoo‐housed groups of geladas (*Theropithecus gelada*, *n*_subjects_ = 67, *n*_yawns_ = 1422) and hamadryas baboons (*Papio hamadryas*, *n*_subjects_ = 28, *n*_yawns_ = 602). The species are optimal candidates as they both form multilevel groups but differ in intra‐group dynamics, cohesion, and cross‐sex bonding. Although both species displayed distinct yawn morphologies, hamadryas yawned less frequently than geladas, mainly in non‐social contexts and without vocalization. In contrast, geladas yawned more often during affiliative interactions, highlighting a more social dimension to their yawns. When focusing on silent yawns, hamadryas showed a male‐biased yawning frequency, whereas geladas exhibited similar rates between sexes, suggesting a more prominent female role in their yawning patterns. We found that yawning is contagious not only in geladas, as previously known, but also in hamadryas baboons. However, geladas were more responsive to others' yawns, possibly due to their greater communicative complexity or to the need to maintain cohesion in larger groups. In geladas, both sexes exhibited similar levels of yawn contagion, whereas in hamadryas it was predominantly male‐driven, reflecting the central role of males in hamadryas social dynamics. Our study suggests both evolutionary inertia and divergence in Papionine yawning evolution. The findings confirm the derived nature of gelada yawn vocalizations and highlight the link between multimodal communication and social complexity. Moreover, geladas exhibit more nuanced, context‐dependent yawning, likely shaped by their intricate sociobiology. In contrast, hamadryas display a more male‐dominated yawning pattern, reflecting their distinct social dynamics. To fully understand the ecological significance of this ancient behavior, further cross‐species research on yawning and its contagious effect in wild populations is essential.

## Summary

- Geladas produce yawn calls, yawn more, and in a larger variety of contexts than hamadryas
- Geladas are more susceptible to others' yawns than hamadryas
- Yawn contagion in hamadryas is mostly shown by males

## Graphical abstract

Geladas produce yawn vocalizations, yawn more, and in a larger variety of contexts than hamadryas. Geladas are more susceptible to others' yawns that hamadryas. Yawn contagion in hamadryas is mostly shown by males. Pictures by V. Maglieri and M. Francesconi.

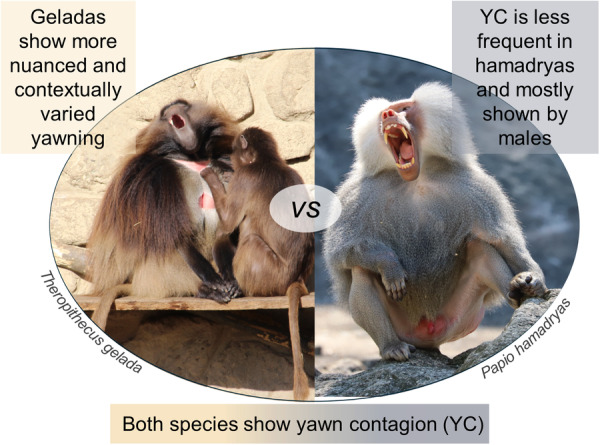

## 1 Introduction

The social complexity hypothesis for animal communication predicts a functional relationship between patterns of communication and patterns of social organizations (Freeberg et al. 2012). Animals forming complex societies are therefore expected to use intricate communication systems to address various social needs (e.g., individual discrimination, expression of emotional states, conveying messages in a variety of contexts) (Peckre et al. 2019). For instance, in mammals as well as birds, derived and rich vocal repertoires seem to have evolved in parallel with species social complexity (Bouchet et al. 2013; Coye et al. 2022; Leighton and Birmingham 2021; Manser et al. 2014; Rebout et al. 2020). However, beyond signal diversity, communicative complexity can also be accomplished by increasing the redundancy (e.g., frequency, duration) and salience (e.g., unimodal vs. bimodal signal) of signals (Fröhlich et al. 2019).

Cross‐species comparisons of highly conserved communicative displays that vary in their morphology or modality can help elucidate the importance of each component in shaping signaling function. Yet, while numerous studies support the social complexity hypothesis by identifying correlations between social and communicative variables, direct comparisons seldom explore how social factors directly influence variation in signaling (Gustison et al. 2012; Manser et al. 2014; Peckre et al. 2019). In vertebrates, one of the most conserved behavioral traits which can assume communicative functions is yawning (Gallup 2022; Moyaho et al. 2017). Yawning likely did not evolve primarily as a communicative signal, as it often occurs in non‐social contexts and is more likely associated with physiological processes like regulating internal homeostasis (Gallup 2022). However, the possible socio‐communicative function of yawning is evident in its contagious nature (Provine 2005), that can have consequences on behavioral synchronization of animal activities (Casetta et al. 2021; Galotti et al. 2024). In mammals, spontaneous yawning is a widespread phenomenon whose function remains partially unknown, possibly related to behavioral and physiological transitions (state changes, Galotti et al. 2024; Vick and Paukner 2010; brain cooling, Gallup 2022; internal state communication, Palagi et al. 2020). Despite being a fixed‐action pattern, yawning expression can vary in terms of morphology and context of production (Leone et al. 2014; Vick and Paukner 2010), frequency and duration (Zannella et al. 2021), and sensory modalities involved (Pedruzzi et al. 2025).

The most complex forms of yawning expression seem to be found in two primate species: humans (*Homo sapiens*) and geladas (*Theropithecus gelada*). Indeed, according to current literature, these are the only species known to produce distinct and intricate vocalizations specifically associated with yawning (Arnott et al. 2009; Palagi et al. 2009), with the sole yawn sound eliciting a contagious response in both species (Arnott et al. 2009; Pedruzzi et al. 2024, 2025). Comparing groups of phylogenetically related species inhabiting social systems with similar structures (e.g., pair‐living, multi‐male multi‐female groups, one‐male groups) but with differences in their sociobiology can yield valuable insights into the possible adaptive role of yawning expression variability.

**Figure 1:**
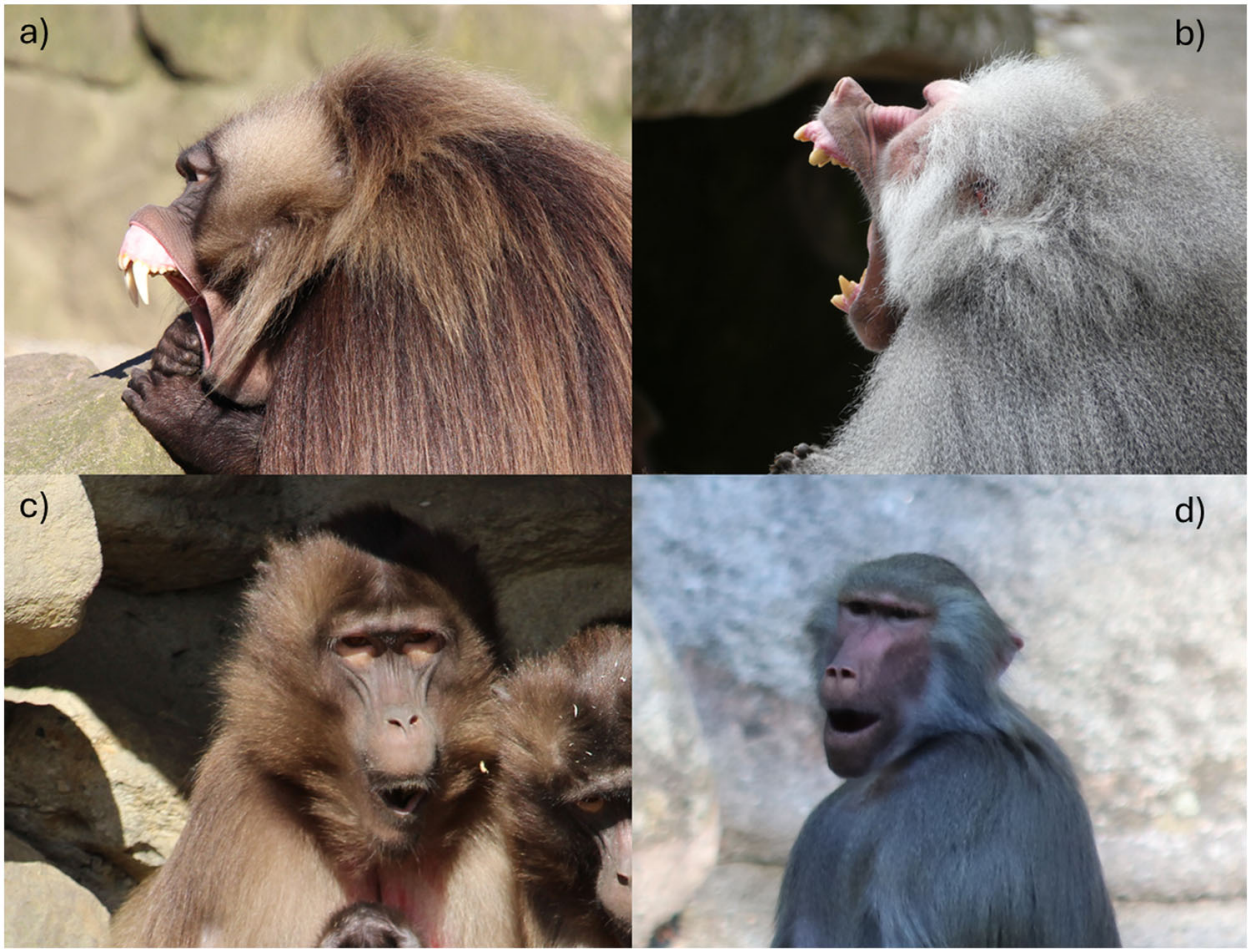
Examples of yawning behavior by males showing gums and teeth in (a) geladas and (b) hamadryas baboons, and by females with covered teeth and gums in (c) geladas and (d) hamadryas. Pictures by V. Maglieri (hamadryas) and M. Francesconi (geladas).

Here, we selected two closely related terrestrial monkey species, geladas (*Theropithecus gelada*) and hamadryas baboons (*Papio hamadryas*), both of which inhabit open environments and live in complex multilevel societies. In these societies, small, stable core groups of individuals come together to form larger social units, creating a hierarchical, nested structure with multiple levels within the population (Grueter et al. 2020). Yet, the two species show significant differences in terms of intra‐unit dynamics and cohesion, cross‐sex bonding, and exogamy (Grueter et al. 2012, 2020; Matsuda et al. 2012). These species are excellent candidates to explore yawning variability: while some aspects suggest evolutionary inertia (different types of yawns, sexual dimorphism, Figure 1), others hint at a rapid divergence (e.g., bimodal yawns for geladas) in the evolution of yawing within Papionine species. However, while yawn contagion has been extensively studied in geladas (Leone et al. 2014; Palagi et al. 2009; Pedruzzi et al. 2024, 2025), no study so far has investigated the phenomenon in baboons. Here, we aimed at exploring the variation in the use (i.e., rate of production, contextual use, sex differences) and structure (i.e., duration, yawn morphology, sensory modality) of yawns as well as in their contagious nature to comprehend the role of acoustic cues in the complexity of yawning expression, but also to unveil how differences in yawning might reflect the differences in the social organizations of the species (Grueter et al. 2012; Snyder‐Mackler et al. 2012).

Geladas show richer and more derived visual (Lazow and Bergman 2020; Palagi and Mancini 2011) and vocal (Gustison et al. 2012; Pedruzzi et al. 2024; Zanoli et al. 2022) communicative repertoires compared to species of the *Papio* genus. Considering this and the fact that species with more diverse repertoire also use their signals more frequently (Bouchet et al. 2013; Freeberg and Harvey 2008), hamadryas are expected to yawn at lower frequencies than geladas (*Prediction 1*), and not to produce distinct vocalizations associated with yawns (*Prediction 2*). Since socially‐complex species also use signals in more varied contexts (Rebout et al. 2022), we expect geladas to have a more diverse spectrum of contexts in which the behavior is shown (*Prediction 3*). As both species show high levels of sexual dimorphism (body and canine size), in both groups males are expected to spontaneously yawn at higher frequencies compared to females (*Prediction 4*), to produce longer (*Prediction 5*) and more yawns exposing their canines (*Prediction 6*), as found in other sexually dimorphic monkeys (Galotti et al. 2024; Troisi et al. 1990; Zannella et al. 2017).

Yawn contagion (YC) is a phenomenon evolved in social species which has been variably described as a form of motor resonance, mimicry or behavioral contagion (Gallup 2022; Massen and Gallup 2017; Palagi et al. 2020). YC can function in enhancing vigilance (Gallup and Meyers 2021), fostering behavioral synchronization (Casetta et al. 2021), inducing shifts in activity states (Galotti et al. 2024), and even strengthening affiliation among group members (Poole and Henderson 2023). Subjects with high levels of motor synchronization or cooperative behavior are likely to be more susceptible to others' yawns (Ake and Kutsukake 2023; Casetta et al. 2021). Moreover, species with loose relationships are expected to show YC at a lesser extent (Palagi et al. 2019). In this view, we expect YC to be present also in the hamadryas group (*Prediction 7*), due to the species complex social dynamics and long‐term interindividual bonds (Swedell et al. 2011). However, due to the more diverse gelada communicative tactics and to their bigger and more complex group units (Gustison et al. 2012; Matsuda et al. 2012), we expect geladas to be more susceptible to others' yawns (*Prediction 8*).

The sociobiology (e.g., intra‐unit dynamics and cohesion, cross‐sex bonding, and exogamy) of wild populations of geladas and hamadryas strongly differs as gelada females are strongly philopatric, remaining in their natal group (Snyder‐Mackler et al. 2012; Tinsley Johnson et al. 2014), whereas hamadryas females are subjected to male‐forced dispersion from their natal group through takeovers (Amann et al. 2017; Pines et al. 2011; Swedell et al. 2011, 2014). Such coerced female grouping in hamadryas leads to relatively low ingroup female‐female kin relationships and bonds (Amann et al. 2017; Matsuda et al. 2012; see also Städele et al. 2016; Swedell 2002). On the other hand, philopatric females in geladas show strong kin relationships and social bonds (Pallante et al. 2019; Tinsley Johnson et al. 2014). In the hamadryas patrilineal society, differently from most baboon species, males are philopatric at the clan and band level, leading to increased male tolerance and cooperation via kin selection with male‐male bonding stronger than female‐female bonding, yet detailed data on the nature and extent of this cooperation remain limited (Evans et al. 2022; Grueter et al. 2012; Romero and Castellanos 2010; Städele et al. 2016). However, direct positive interactions between males from different group units are rare (Swedell and Schreier 2009), meaning that within‐group male‐female interactions are surely stronger than between‐group male‐male interactions also for hamadryas (Grueter et al. 2012). In geladas, intermale interactions are rarer compared to hamadryas (Grueter et al. 2012). Due to such sex differences in social bonding and cooperation, gelada females should be more central in YC, while hamadryas males are predicted to be more responsive to yawns in general, regardless of the yawner's sex (*Prediction 9*).

## 2 Methods

### 2.1 Ethics Statement

The study involved recording geladas and hamadryas baboons from a distance, with no direct contact or manipulation of the animals. This recording process adhered to American Society of Primatologists Principles for the Ethical Treatment of Nonhuman Primates (e.g., maintaining an appropriate recording distance, avoiding any kind of distress for the animals). Consequently, the ethics committee of the University of Pisa waived the requirement for a permit.

### 2.2 Study Groups, Data Collection, Video Analyses

#### 2.2.1 Theropithecus gelada

Data collection occurred at NaturZoo Rheine (Germany) in April–May 2023, 7 days a week (8:30 a.m.–1 p.m.; 3 p.m.–8 p.m.). NaturZoo Rheine houses the world's largest captive gelada colony (social housing condition: Continuous Full Contact, group), consisting of 103 individuals (Pedruzzi et al. 2025). The colony was divided into two enclosures (G1 and G2, Table S1). Following the EEP gelada program guidelines to prevent inbreeding, the adult males and some subadults from G2 were relocated to other zoos. Data from G2 collected after the males' removal were excluded to minimize differences between the gelada and hamadryas baboon groups. The gelada enclosure featured indoor spaces (36 m²) and a large outdoor area (2700 m²) where the animals could move freely. They were fed twice daily (9:30 a.m., 2:30 p.m.) with grass, vegetables, and pellets, and water was always available. All study subjects, including all adults and most subadults in the group, were individually identified.

#### 2.2.2 Papio hamadryas

Data collection took place in June–September 2019 (5 days a week, 9 a.m.–12 p.m., 2 p.m.–7 p.m.) at Hellabrunn Munich Zoo (Germany), where a colony of 29 subjects (15 males, 14 females) is hosted (Table S1) (social housing condition: Continuous Full Contact, group). The enclosure (1500 m^2^) had both indoor and outdoor areas, both freely accessible by the animals. The outer area consisted of concrete, rock, soil, trees, poles, and was enriched with rigid tapes and suspended ropes. Food was provided twice a day, (between 7:30 a.m.–9:30 a.m. and 1:30 p.m.–4 p.m.). Water was always available. The study subjects (all adults) were individually identified.

Three observers (L.P., P.O., M.F., using SONY Handycam Full HD FDR‐AX43A, accuracy 60 fps, with directional microphones Sennheiser MKE600) spread in different sections of the enclosure recorded the gelada colony, whereas two (R.R., F.B., using Panasonic HC‐V180 Full HD, accuracy 60fps, with microphones Sennheiser MKE600) recorded the hamadryas colony. Although slight variations in video frame size due to zoom adjustments cannot be ruled out, we generally maintained a consistent zoom setting to capture all subjects within approximately 10 m of the trigger yawner. Using all‐occurrences sampling (Altmann 1974), we recorded social interactions (e.g., affiliation, proximity, yawning events) by randomly following subgroups visible to the observers. We collected 230 h of recordings for the gelada colony and 123 h for hamadryas, calculating individual recording time through 3‐min scan sampling during video analysis. For each animal, we summed the scans in which the subject was present and derived a proxy for its recording time by multiplying this number by three (e.g., 240 scans = 720 min of observation) (Pedruzzi et al. 2025) (mean individual recording time ±SE: geladas: 5.80 ± 1.40 h; hamadryas baboons: 4.58 ± 1.55 h). Videos were analyzed using PotPlayer to precisely record the occurrence and duration of specific behavioral patterns. For each yawn, we noted the exact time, the yawner's identity, the morphological variant (yawn type commonly present in monkeys: yawns with covered teeth, Y1, with uncovered teeth but covered gums, Y2, and with uncovered teeth and gums, Y3, Palagi et al. 2009), whether it was vocalized, its duration, the identity and number of nearby subjects, whether the yawn was detected by any receivers, and the behavioral context involving the yawner in the 5 s before the yawn (see definitions below).

## 3 Operational Definitions

### 3.1 Visual Detection

We coded if yawns were visually detected by each receiver subject. Yawns were coded as not visually detected (*Not seen*) when the face of the receiver turned 180° away from the trigger or when any obstacle prevented the receiver from seeing the trigger subject, as geladas and hamadryas baboons, like most primates, are characterized by high orbit convergence and large binocular visual field (Heesy 2004). Otherwise, yawns were coded as visually detected (*Seen*) if the trigger yawn was in the visual field of the receiver. All doubtful cases (74 on 2972 trigger‐receiver cases) were excluded.

### 3.2 Response

We classified as yawn responses any yawns made by receiver subjects within 3 min of the trigger yawn. This 3‐min time frame was chosen based on existing research on yawn contagion in nonhuman primates (Campbell and Cox 2019; Palagi et al. 2009; Pedruzzi et al. 2025). Subjects that were not visible or could not be continuously recorded during the entire 3‐min period after the trigger yawn (e.g., due to moving out of the observers' range) were excluded from the analysis. Additionally, receivers exposed to multiple trigger yawns from different individuals before yawning were excluded due to the difficulty in determining which trigger yawn prompted their response (geladas, *n* = 83 cases, hamadryas, *n* = 33 cases). *Number of subjects in the audience*. We coded for the number of subjects present in the audience (*Audience size*) for every trigger yawn (i.e., within a range of about 10 m from the trigger yawner).

### 3.3 Grooming Index and Proximity Index

To assess the strength of relationships between two individuals, we calculated grooming and proximity indices for all possible dyads in the two groups. These indices were derived using the scan‐sampling method, where every 3 min, we identified all subjects visible in the video frame and recorded their grooming and proximity behaviors. The *Grooming Index* was calculated by dividing the number of scans in which two individuals groomed each other (regardless of grooming direction) by the total number of scans in which both individuals were present. The *Proximity Index* was calculated by dividing the number of scans in which an individual was within proximity (defined as two individuals sitting no farther apart than the length of an outstretched limb) with a specific individual by the number of scans in which both individuals were present. We included both indices because spatial proximity is not always a good proxy for social bonding (Tinsley Johnson et al. 2014). Additionally, by accounting for proximity levels between subjects, we can determine whether yawn contagion is influenced by varying degrees of social bonding or detection probability (Gallup 2021).

### 3.4 Individual (ID) Frequency of Spontaneous Yawning

For each subject, we calculated the individual spontaneous yawn frequency (yawns/minute) as follows: number of spontaneous yawns (derived from the total yawns performed by an individual minus those emitted following the detection of others' yawns in a time‐window of 3 min) divided by the recording time for the individual.

### 3.5 Main Context Before Yawning

To determine the main behavioral context preceding yawning, we coded the context in which the yawner was during the 5 s before yawning. The choice of this timeframe was based on a reasonable assumption that it would best capture the immediate conditions leading to a yawn. While unfortunately no specific standard exists in the literature, we selected this window as a practical and intuitive measure of the preceding behavioral context. The contexts recorded could either be: (i) affiliation (e.g., grooming or being groomed, hugging behavior), (ii) post‐aggression period, (iii) non‐social neutral context (e.g., resting, laying down, sitting alone), (iv) post‐copulation, and (v) self‐directed behaviors. When more than one context was present (e.g., laying down and being groomed or being in a post‐conflict period and resting), we defined a hierarchy of context importance, mainly based on the level of arousal and social value associated with the context, to determine the main context of yawning: (i) aggression, (ii) post‐copulation, (iii) affiliation, (iv) self‐directed behaviors, (v) neutral non‐social context.

### 3.6 Inter‐Coder Agreement

Inter‐coder reliability between LP and PO (gelada group) and between V.M. and R.R. (hamadryas group) was assessed independently on approximately 15% of the videos using Cohen's *κ* coefficient (Cohen 1968), consistently achieving a value greater than 0.80 (geladas: subject identification, *K* = 0.86; yawn presence, yawn type, modality, *K*_average_ = 0.90; detection *K* = 0.87; grooming and proximity, *K*_average_ = 0.97; context, *K* = 0.98; hamadryas baboons: subject identification, *K* = 0.89; yawn presence and type, *K*_average_ = 0.93; visual detection *K* = 0.91; grooming and proximity, *K*_average_ = 0.95; context *K* = 0.96).

## 4 Statistical Analyses

All analyses were conducted using RStudio (http://www.r-project.org).

To test our predictions, we employed a series of Generalized Linear Mixed Models (GLMMs) examining different aspects of yawning behavior. We first analyzed spontaneous yawning frequency, yawn duration, and yawn morphology in relation to species and sex (Models 1–3, testing Prediction 1, 4–6). We then investigated the context of yawn production, first running a model to compare the yawn morphology according to sex and species in neutral non‐social context for both species (Model 4, testing Prediction 3), and further exploring the use of different yawn types within geladas during both affiliative and neutral contexts (Model 5, testing Prediction 3). Finally, we examined yawn contagion, assessing individuals' responses to others' yawns and the factors influencing yawn contagion across species and sexes (Models 6–7, testing Prediction 7–9). Since only geladas produced yawns associated with a vocalization (see Results), we ran models either excluding (Model 1a, 2a, 3a, 4a, 5a, 6, 7a) and including (Model 1b, 2b, 3b, 4b, 5b, 6, 7b) vocalized yawns.

Multicollinearity in the GLMMs was assessed via the check_collinearity function from the performance package (version 0.4.4) using Variance Inflation Factors (VIFs). All fixed factors in the models showed low correlation (VIF: 1.09–2.90). Model significance was evaluated by comparing the full model to a null or control model, which included only random effects (and control factors), using the Likelihood Ratio Test (LRT) with the Chisq test argument (Dobson and Barnett 2018). To determine the *p*‐value of predictors, LRTs were performed between the full model and a model without the specific predictor, using the ANOVA function (Barr et al. 2013). Marginal and conditional *R* ^2^ values were calculated with the MuMIn package (version 1.43.17) (Barton 2020). For pairwise comparisons involving factors with more than two levels (and for interaction factors), the emmeans package was used to perform the Tukey test (Bretz et al. 2016; Lenth 2025). Model fit and potential overdispersion were assessed with the DHARMa package (version 0.3.3.0) (Hartig 2020). The GLMMs showed no overdispersion (dispersion range: 0.14–0.77, *p*‐value range: 0.98‐1), no outliers were detected (*p*‐value range: 0.07–1), and the normality of residuals was confirmed through visual inspection of Q‐Q plots (Kolmogorov–Smirnov test, *p*‐value range: 0.06–0.81).

### 4.1 Spontaneous Yawning: Frequency, Yawn Duration, and Yawn Type According to Sex and Species

**Spontaneous frequency according to sex and species: without vocalized yawns (Model 1a); with vocalized yawns (Model 1b)—*Prediction 1, 4*. Model 1a.** To test Prediction 1 and 4, we ran a GLMM with the *Spontaneous yawning frequency* (yawns/minute, 0 < frequency < 1) of each subject as response variable using a beta distribution for proportion data. The fixed factors considered were: (i) *Species*, (ii) *Sex*, and the (iii) interaction *Species* *Sex. **Model 1b**. Apart from the inclusion of vocalized yawns, Model 1b was built as Model 1a.

**Yawn duration according to sex, species, yawn morphology: without vocalized yawns (Model 2a); with vocalized yawns (Model 2b)—*Prediction 5*. Model 2a.** To test Prediction 5, we ran a GLMM for all scored yawns with *Yawn duration* (seconds, log‐transformed) as response variable using a Gaussian distribution applied after log‐transformation and the exclusion of outliers. The *Subject ID* was included as random factor, and the fixed factors considered were: (i) *Species*, (ii) *Sex*, (iii) *Yawn type* (type 1/type 2/type 3), as well as (iv) the interactions Species*Sex*Yawn type. **Model 2b.** Apart from the inclusion of vocalized yawns, Model 2b was built as Model 2a.

**Yawn morphology according to sex and species: without vocalized yawns (Model 3a); with vocalized yawns (Model 3b)—*Prediction 6*. Model 3a.** To understand the distribution of the three yawn morphological variants and to test Prediction 6, we built a data set with each line being the total number of yawns recorded for each unique combinations of species, type, sex, and yawner. We ran a GLMM with the *Number of yawns produced* as a response variable (negative binomial distribution for count data due to overdispersion when applying Poisson distribution). The *Subject ID* was included as random factor and the *Individual spontaneous yawn frequency* as a control factor; the fixed factors considered were: (i) *Species*, (ii) *Sex,* (iii) *Yawn type* and (iv) the interactions Species*Sex*Yawn type. **Model 3b.** Apart from the inclusion of vocalized yawns, the model was built as Model 3a.

### 4.2 Context of Yawn Production According to Sex and Species

Hamadryas baboons hardly ever yawned in affiliative/post‐copulative contexts, and we recorded relatively few yawns after aggression. Thus, we focused our analyses on yawning during affiliative (geladas) and non‐social neutral contexts (both groups) (see Results for details). The context of allogrooming was present in both groups, as in about 74% of the 3‐min scans done on all gelada videos collected at least 1 dyad was engaging grooming, and even in 93% of scans for hamadryas baboons.

**Yawning in neutral context: morphology use according to sex and species: without vocalized yawns (Model 4a); with vocalized yawns (Model 4b)—*Prediction 3*. Model 4a.** We built a data set with each line being the total number of yawns recorded only those produced in neutral context for each unique combinations of species, type, and sex, per each yawner. To test Prediction 3, we ran a GLMM with the *Number of yawns produced* as a response variable using a negative binomial distribution. The *Subject ID* was included as random factor and the *Individual spontaneous yawn frequency* as control factor; the fixed factors considered were: (i) *Species*, (ii) *Sex,* (iii) *Yawn type* and (iv) the interactions Species*Sex*Yawn type. **Model 4b.** Apart from the inclusion of vocalized yawns, the model was built as Model 4a.

**Yawning during affiliative and neutral context in geladas: morphology use according to sex and context, without vocalized yawns (Model 5a); with vocalized yawns (Model 5b)—*Prediction 3*. Model 5a.** To understand the different use of the three yawn types by males and females of the gelada colony in affiliative and non‐social context, testing Prediction 3, we built a data set with each line being the total number of yawns recorded for each unique combinations of type, sex per each yawner and, importantly, normalized for the frequency of non‐social and affiliative context in the gelada colony. We ran a GLMM with the *Number of yawns produced* as a response variable using a negative binomial distribution. The *Subject ID* was included as random factor and the *Individual spontaneous yawn frequency* as a control factor; the fixed factors considered were: (i) *Context*, (ii) *Sex,* (iii) *Yawn type* and (iv) the interactions Context*Sex*Yawn type. **Model 5b.** Apart from the exclusion of vocalized yawns, Model 5b was built as Model 5a.

### 4.3 Contagious Yawning

**Model 6. Responding to others' yawns: yawn contagion in the two groups—*Prediction 7, 8, 9*.** To test Prediction 7 to 9, we ran a GLMM with *Yawn response* as response variable (presence/absence) using a binomial error distribution, with each model observation being a trigger‐receiver pair for each yawning event. The interaction between the identity of *Trigger* and *Receiver* subjects and the *Trigger yawn ID*s were included as random factor; *Individual spontaneous yawn frequency*, Proximity index*Species, and Audience size*Species were included as control factor. The fixed factors considered were: (i) *Visual detection* (No/Yes), (ii) Trigger sex*Receiver sex, (iii) *Grooming index*, (iv) *Type of trigger yawn*, (vi) *Duration of trigger yawn* (seconds), as well as the interactions: (i) Visual detection*Species (gelada/hamadryas baboon), (ii) Trigger sex*Receiver sex*Species, (iii) Grooming index*Species.

**Yawn contagion index according to species and sex: without vocalized yawns (Model 7a); with vocalized yawns (Model 7b)—*Prediction 7, 8, 9*. Model 7a.** To further test Prediction 7 to 9, we ran a GLMM with the *Yawn contagion index* of each study subject as response variable using a beta model for proportions. The index was calculated by dividing the times a subject yawned after detecting others' yawns (within 3 min, cases of yawn contagion) on the total number of yawns perceived, including yawns in response to vocalized yawns. The subjects who never showed spontaneous yawning were excluded, as were the subjects who perceived less than three yawns during the data collection. The *subject ID* was included as random factor; the *Number of yawns perceived* and the *Individual spontaneous yawn frequency* were included as control factor. The fixed factors considered were: (i) *Species*, (ii) *Sex*, and (iii) the interaction Species*Sex. **Model 7b.** Apart from the exclusion of vocalized yawns, the model was built as Model 7a.

## 5 Results

### 5.1 Spontaneous Yawning: Frequency, Yawn Duration, and Yawn Type According to Sex and Species

We collected a total of 2024 yawns (*n* = 1422 by 67 geladas; *n* = 602 by 28 hamadryas baboons). We only recorded distinct yawn‐associated vocalizations and lip‐flip movements in geladas (*Prediction 2* supported). Since only geladas produced yawns (*n* = 495) associated with a vocalization, we provide in the following analyses models comparing the exclusion and inclusion of such multimodal yawns to test the hypotheses.

**Figure 2:**
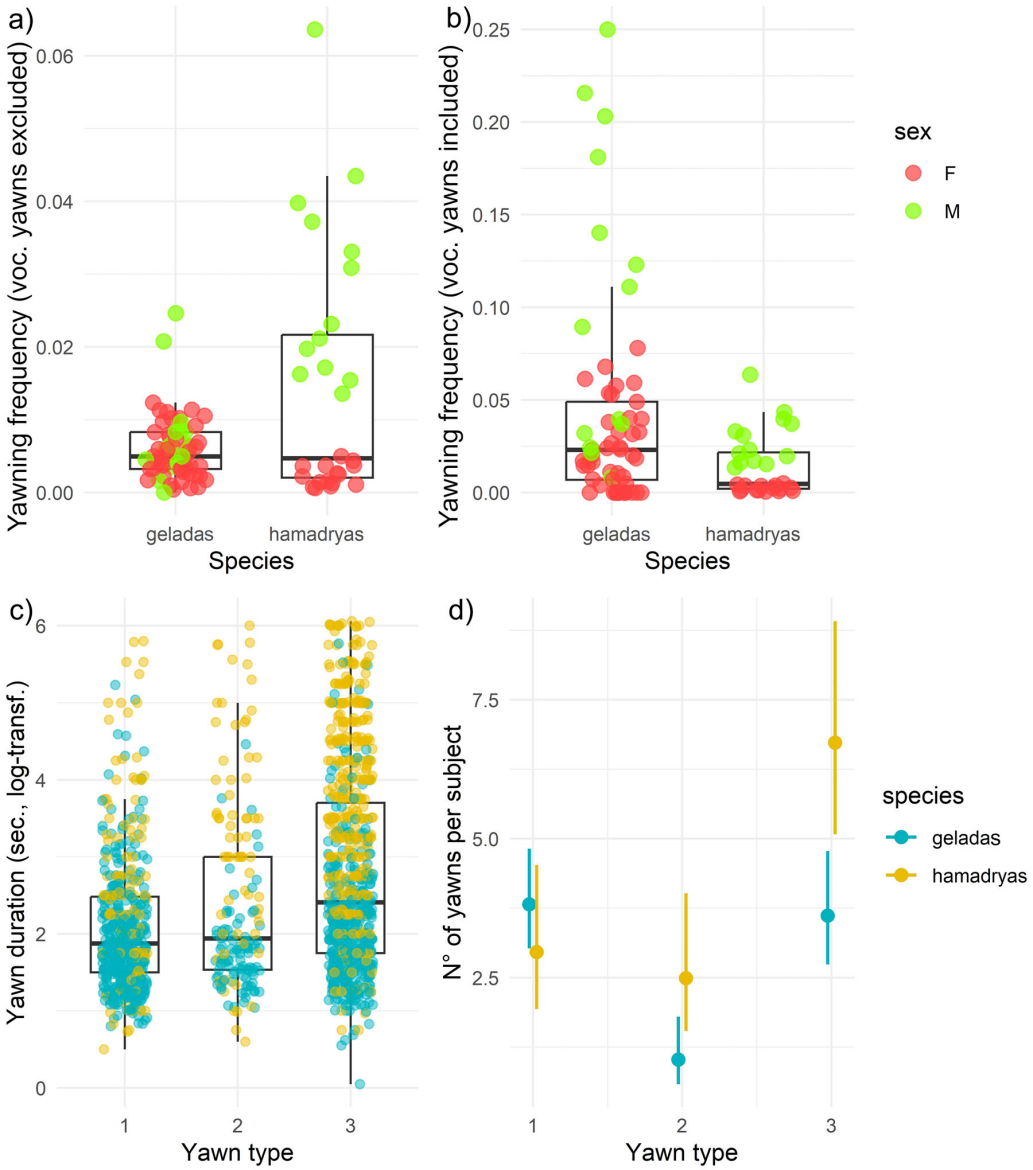
Boxplots comparing the frequency of yawning (number of spontaneous yawns/recording time) in geladas vs. hamadryas baboons (a) excluding or (b) including gelada vocalized yawns; each plotted dot represent a subject (red = F, female; green = M, male); (c) boxplots comparing the duration of yawns (seconds) according to the yawn type; each plotted dot represents a yawn (yellow = hamadryas; blue = gelada); the boxes display the median value and first and third quartiles, whiskers are extended to the most extreme value inside the 1.5‐fold interquartile range; (d) effect plot showing the significant interaction Species*Yawn type on the number of yawns recorded in Model 3a (yellow line = hamadryas; blue line = gelada), error bars represent confidence intervals.

**Spontaneous frequency according to sex and species: without vocalized yawns (Model 1a); with vocalized yawns (Model 1b)—*Prediction 1, 4*. Model 1a.** Number of observations = 88, with subjects who never yawned or who only yawned vocalizing were excluded (*n* = 7). Sex*Species significantly affected the spontaneous yawning frequency (*χ* ^2^ = 39.374, *p* < 0.0001, Table 1a). Gelada males and females had comparable spontaneous yawning frequencies (*β* = −0.388, *p* = 0.12, Figure 2a), whereas hamadryas males yawned more frequently than gelada males (*β* = −1.232, *p* < 0.0001, Figure 2a), gelada females (*β* = −1.620, *p* < 0.0001, Figure 2a), and hamadryas females (*β* = −2.160, *p* < 0.0001, Figure 2a) (*Prediction 4* partially supported, *Prediction 1* not supported). **Model 1b.** Number of observations = 89, with subjects who never yawned excluded (*n* = 6). Males spontaneously yawned more frequently than females (*χ* ^2^ = 52.88, *p* < 0.0001, Table 1b), and geladas yawned more than hamadryas baboons (*χ* ^2^ = 6.753, *p* = 0.0094, Figure 2b), whereas the interaction Sex*Species was not significant (*Prediction 1* and *4* supported).

**Table 1:** Estimated prameters (Coeff), Standard Error (SE), and results of the Likelihood Ratio Tests (*χ* ^2^) of the GLMMs.

Fixed effects	Coeff	SE	*χ* ^2^	df	*p*
a. *Model 1a—Spontaneous frequency (vocalized yawns excluded)—Prediction 1, 4*					
Intercept	−5.190	0.102	—	—	—
**Tested variables**					
Sex (Male)	0.388	0.176	58.628	1	**0.000**
Species (Hamadryas baboon)	−0.540	0.220	16.001	1	**0.000**
Sex*Species	1.772	0.282	39.374	1	**0.000**
*N*_observations_ = 88					
b. *Model 1b—Spontaneous frequency (vocalized yawns included)—Prediction 1, 4*					
Intercept	−3.921	0.176	—	—	—
**Tested variable**					
Sex (Male)	1.526	0.230	52.890	1	**0.000**
Species (Hamadryas)	−0.335	0.282	6.753	1	**0.009**
Sex*Species	−0.363	0.396	0.841	1	0.359
*N*_observations_ = 89					
c. *Model 2a—Yawn duration (vocalized yawns excluded)—Prediction 5*					
Intercept	0.638	0.030	—	—	—
**Tested variables**					
Sex	‐0.111	0.056	2.484	1	0.115
Species	0.207	0.084	124.944	1	**0.000**
Type	—	—	82.398	2	**0.000**
Type 2	0.033	0.111	—	—	—
Type 3	0.134	0.041	—	—	—
Sex*Species	0.148	0.110	1.417	1	0.234
Sex*Type	—	—	0.036	2	0.982
Male:Type 2	0.032	0.126	—	—	—
Male:Type 3	0.026	0.061	—	—	—
Species*Type	—	—	18.747	2	**0.000**
Hamadryas:Type 2	0.232	0.154	—	—	—
Hamadryas:Type 3	0.288	0.093	—	—	—
Sex*Species*Type	—	—	0.556	2	0.757
Male:Hamadryas:Type 2	−0.106	0.181	—	—	—
Male:Hamadryas:Type 3	−0.078	0.116	—	—	—
*N*_observations_ = 1398, N_subjects_ = 97. Random factors: Subject ID, Variance = 0.013, SD = 0.114.					
d. *Model 3a—Yawn morphology (vocalized yawns excluded)—Prediction 6*					
Intercept	1.279	0.126	—	—	—
**Tested variables**					
Sex	−0.684	0.270	6.602	1	**0.010**
Species	−0.753	0.333	1.640	1	0.200
Type	—	—	52.548	2	**0.000**
Type 2	−1.259	0.365	—	—	—
Type 3	−0.287	0.158	—	—	—
Sex*Species	—	—	20.437	1	**0.000**
Sex*Type	—	—	9.287	2	**0.009**
Male:Type 2	−0.155	0.584	—	—	—
Male:Type 3	0.625	0.364	—	—	—
Species*Type	—	—	14.126	2	**0.000**
Hamadryas:Type 2	1.067	0.584	—	—	—
Hamadryas:Type 3	0.805	0.382	—	—	—
Sex*Species*Type	—	—	0.136	2	0.934
Male:Hamadryas:Type 2	0.207	0.787	—	—	—
Male:Hamadryas:Type 3	0.191	0.546	—	—	—
**Control variable(s)**					
Individual spontaneous yawn frequency	8.379	1.983	17.861	1	0.000
*N*_observations_ = 161, *N*_subjects_ = 86. Random factors: Subject ID, Variance = 0.120, SD = 0.346.					

*Note:* Significant *p* values are in bold; df = degree(s) of freedom; − = not applicable. Estimate ±SE refers to the difference of the response between the reported level of this categorical predictor and the reference category of the same predictor.

**Yawn duration according to sex, species, yawn morphology: without vocalized yawns (Model 2a); with vocalized yawns (Model 2b)—*Prediction 5*. Model 2a.** Number of observations = 1398, with all vocalized yawns and those with unclear yawn type excluded. Random factor: *Yawner ID* = 97. The full model significantly differed from the null one (*χ* ^2^_11_ = 190.87, *p* < 0.0001). The interaction Yawn type*Species (*χ* ^2^ = 18.747, *p* < 0.0001, Table 1c) significantly affected the duration of yawns. Specifically, results from the Tukey test (df = 1384) showed that hamadryas type 1 yawns were longer than gelada type 1 yawns (*t*‐ratio = −5.084, *p* < 0.0001; Figure 2c), hamadryas type 2 yawns were longer than gelada type 2 yawns (*t*‐ratio = −5.501, *p* < 0.0001; Figure 2c), and hamadryas type 3 yawns were longer than gelada type 3 yawns (*t*‐ratio = −11.756, *p* < 0.0001; Figure 2c). Moreover, *Yawn type* differently affected the yawn duration for the *Species* as for hamadryas type 3 yawns were longer than type 2 (*t*‐ratio = −3.037, *p* = 0.029), type 2 yawns were longer than type 1 (*t*‐ratio = −3.514, *p* = 0.0061), and type 3 were longer than type 1 yawns (*t*‐ratio = −8.057, *p* < 0.0001, Figure 2c), whereas for geladas type 3 yawns were longer than type 1 yawns (*t*‐ratio = −4.866, *p* < 0.0001, Figure 2c) and the other yawn types did not differ in their duration (type 1 vs. type 2, *t*‐ratio = −0.788, *p* = 0.970; type 2 vs. type 3, *t*‐ratio = −1.569, *p* = 0.619; Figure 2c). The *Sex* of the yawner and the other fixed factors did not affect yawn duration (*Prediction 5* not supported, Table 1c). **Model 2b.** Number of observations=1779. Random factor: *Yawner ID* = 102. The full model differed from the null one (*χ* ^2^_11_ = 223.06, *p* < 0.0001). The interaction Yawn type*Species (*χ* ^2^ = 17.93, *p* = 0.0001, Table 1d) significantly affected the duration of yawns. The significance of the comparison for each level did not change compared to Model 2a (*Prediction 5* not supported, Table S2a).

**Yawn morphology according to sex and species: without vocalized yawns (Model 3a); with vocalized yawns (Model 3b)—*Prediction 6*. Model 3a.** Number of observations = 238 unique combinations of species, sex, yawn type, vocalized yawns excluded. Random factor: *Yawner ID* = 106. The full model significantly differed from the null one (*χ* ^2^_11_ = 101.71, *p* < 0.0001). The number of yawns produced was affected by *Yawn type*, *Sex* and by the interaction Yawn type*Sex (*χ* ^2^ = 9.29, *p* = 0.096, Table 1e), Species*Sex (*χ* ^2^ = 20.44, *p* < 0.0001, Table 1e), Species*Yawn type (*χ* ^2^ = 14.13, *p* = 0.0009, Table 1e). Males produced more type 3 yawns compared to females (Tukey test results, male vs. female, estimate = −0.70, *p* = 0.03, Figure 2d), and more type 2 and type 3 yawns compared to type 1 yawns (Tukey test, for males, type 1 vs. type 2, *β* = 0.777, *p* = 0.038; type 2 vs. type 3, *β* = −1.613, *p* < 0.0001; type 1 vs. type 3, *β* = −0.836, *p* = 0.002, Figure 2d). Hamadryas males produced more yawns than females (*β* = −0.941, *p* = 0.038), whereas no difference was found between gelada males and females (*β* = 0.302, *p* = 0.30) (*Prediction 6* partially supported). Hamadryas produce more type 3 yawns compared to geladas (*β* = −0.815, *p* = 0.005) and compared to other morphologies (type 2 vs. type 3, *β* = −1.092, *p* < 0.0001; type 1 vs. type 3, β = ‐0.926, *p* < 0.0001), whereas geladas produced more type 1 and type 3 yawns compared to type2 (type 1 vs. type 2, *β* = 1.336, *p* = 0.0001; type 2 vs. type 3, β = −1.362, *p* < 0.0001). The *Species* and the other interaction combinations between the fixed factors did not affect yawn morphology (Table 1e). **Model 3b.** Number of observations, including vocalized yawns = 251. Random factor: *Yawner ID* = 106. The full model differed from the null one (*χ* ^2^_11_ = 98.44, *p* < 0.0001). As in Model 3a, also including vocalized yawns significant fixed factors remained *Yawn type*, *Sex* and by the interaction Yawn type*Sex (*χ* ^2^ = 15.50, *p* = 0.0004, Table S2b), Species*Sex (*χ* ^2^ = 13.86, *p* = 0.0002, Table 1f), Species*Yawn type (*χ* ^2^ = 13.30, *p* = 0.0013, Table S2b). The significance of the comparison for each level did not change compared to Model 3a (*Prediction 6* partially supported).

### 5.2 Context of Yawn Production According to Sex and Species

**Figure 3:**
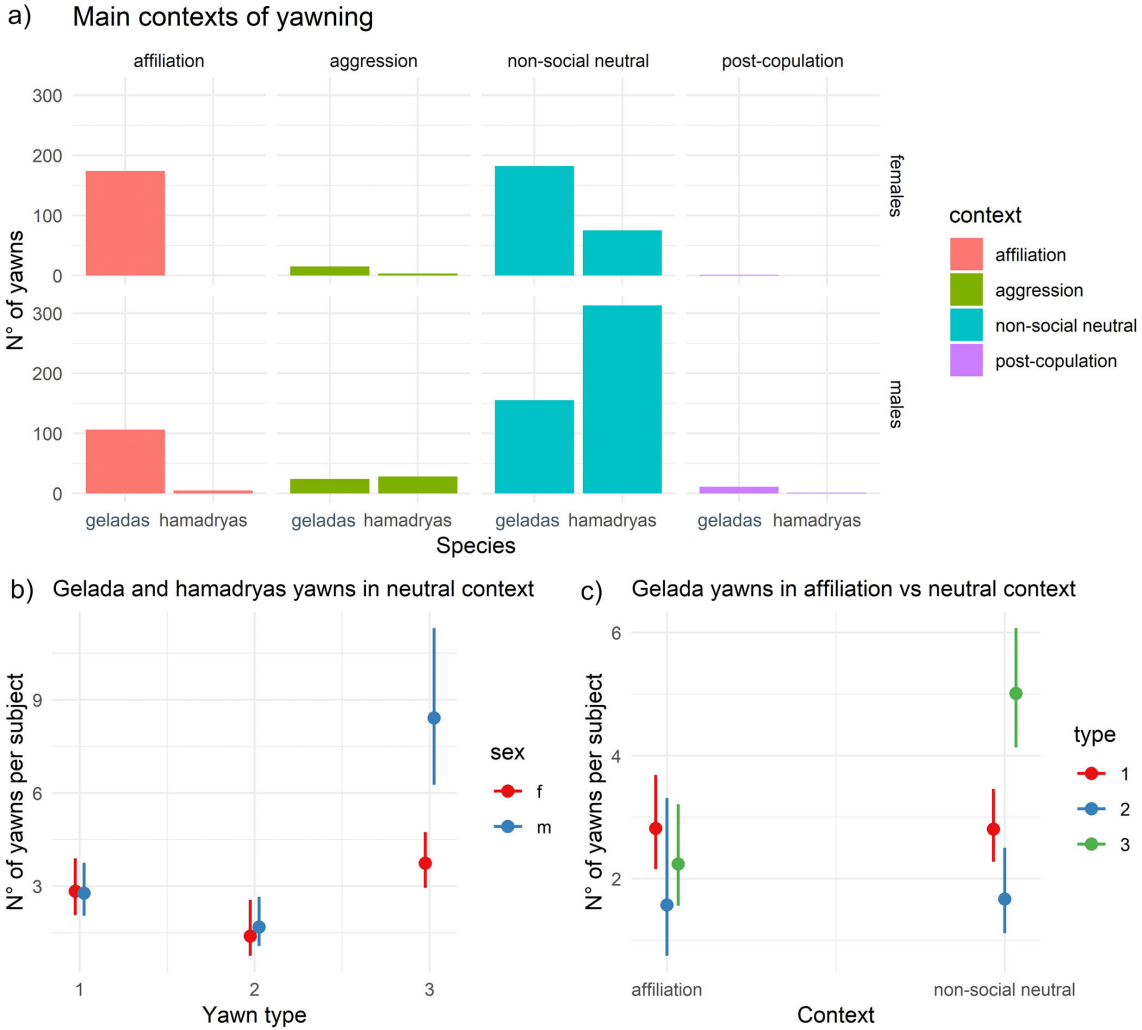
(a) Representation of the main contexts of yawning according to the species and sex of the yawners; (b) effect plot showing the significant interaction Yawn type*Sex on the number of yawns recorded in Model 4a (red line = f, female; blue line = m, male), (c) effect plot showing the significant interaction Yawn type*Context on the number of yawns recorded in Model 5a (red line = type 1 yawns; blue line = type 2 yawns; green line = type 3 yawns); error bars represent confidence intervals.

Figure 3a shows the descriptive distributions of recorded yawns in different contexts. The contexts of production of the recorded yawns (see Operational definitions) were: affiliation (e.g., grooming or being groomed, sit in contact, hugging), post‐aggression period (yawning in the 3‐min window after an aggression), post‐copulation (only males yawned after mating), and neutral non‐social context (e.g., resting, lying down, sitting). Hamadryas rarely yawned in the affiliative context (*n* = 4 yawns), only geladas produced post‐copulation yawns (*n* = 13), and we rarely recorded yawns after aggression (*n* = 32 in hamadryas, *n* = 38 in geladas); thus, we focused next analyses on the yawn production during affiliative (for the gelada group, *n* = 284) and non‐social neutral (for both groups, *n* = 715 yawns) context (*Prediction 3* supported).

**Yawning in neutral context: morphology use according to sex and species: without vocalized yawns (Model 4a); with vocalized yawns (Model 4b)—** ***Prediction 3*** **supported. Model 4a.** Number of observations = 142 unique combinations of species, sex, yawn type for each yawner. Random factor: *Yawner ID* = 75. The full model significantly differed from the null one (*χ* ^2^_11_ = 109.43, *p* < 0.0001). The number of yawns produced in the neutral context, normalized for context occurrence, was significantly affected by *Species, Yawn type*, *Sex*, Yawn type*Sex, and Sex*Species (Table 2a). Hamadryas baboons produced more yawns compared to geladas in neutral non‐social context (*χ* ^2^ = 60.574, *p* < 0.0001). Then, males of both groups produced more type 3 yawns compared to females (Tukey test results, type 3 yawns in geladas, male vs. female, *β* = −804, *p* = 0.003; Figure 3b) and compared to the other morphologies (type 2 vs. type 3, *β* = −1.617, *p* < 0.0001; type 1 vs. type 3, *β* = −1.022, *p* < 0.0001, Figure 3b). As in Model 3a and 3b, hamadryas males produced more yawns than females such difference was found was not present in the group of geladas (Table 2a). **Model 4b.** Number of observations = 146 unique combinations of species, sex, yawn type for each yawner also including vocalized yawns. Random factor: *Yawner ID* = 76. Again, the full model differed from the null one (*χ* ^2^_11_ = 113.61, *p* < 0.0001) and the significant fixed factors were *Species, Yawn type*, *Sex*, Yawn type*Sex, and Sex*Species (Table S3a).

**Table 2:** Estimated parameters (Coeff), Standard Error (SE), and results of the Likelihood Ratio Tests (*χ* ^2^) of the GLMMs.

Fixed effects	Coeff	SE	*χ* ^2^	df	*p*
a. *Model 4a. Yawning in neutral context (vocalized yawns excluded)*					
Intercept	1.279	0.126	—	—	—
**Tested variables**					
Species (Hamadryas)	−0.760	0.331	1.686	1	0.194
Sex (Male)	‐0.693	0.269	6.690	1	**0.009**
Type	—	—	53.439	2	**0.000**
Type 2	−1.265	0.364	—	—	—
Type 3	−0.294	0.157	—	—	—
Sex*Type	—	—	9.442	2	**0.009**
Male:Type 2	−0.144	0.581	—	—	—
Male:Type 3	0.639	0.361	—	—	—
Species*Type	—	—	14.094	2	**0.000**
Hamadryas:Type 2	1.073	0.581	—	—	—
Hamadryas:Type 3	0.813	0.380	—	—	—
Sex*Species	1.364	0.444	20.821	1	**0.000**
Sex*Species*Type	—	—	0.095	2	0.954
Male:Hamadryas:Type 2	0.175	0.783	—	—	—
Male:Hamadryas:Type 3	0.157	0.541	—	—	—
**Control variable(s)**					
Individual spontaneous yawn frequency	8.346	1.970	17.955	1	0.000
*N*_observations_ = 161, N_subjects_ = 86. Random factors: Subject ID, Variance = 0.120, SD = 0.346.						
*b. Model 5a. Yawning during affiliative and neutral context in geladas* (*vocalized yawns excluded*)*.*					
Intercept			—	—	—
**Tested variables**					
Sex (Male)	−0.699	0.321	15.083	2	**0.000**
Context (Non‐social neutral)	−0.344	0.176	3.339	1	0.067
Type	—	—	48.087	2	**0.000**
Type 2	−0.671	0.506	—	—	—
Type 3	−0.587	0.244	—	—	—
Context*Sex	0.913	0.359	14.327	1	**0.000**
Sex*Type	—	—	23.767	2	**0.000**
Male:Type 2	0.236	0.805	—	—	—
Male:Type 3	0.958	0.474	—	—	—
Context*Type	—	—	10.969	2	**0.004**
Non‐social neutral:Type 2	−0.020	0.593	—	—	—
Non‐social neutral:Type 3	0.761	0.295	—	—	—
Context*Sex*Type	—	—	0.101	2	0.950
Male: Non‐social neutral:Type 2	0.288	0.892	—	—	—
Male: Non‐social neutral:Type 3	0.133	0.530	—	—	—
**Control variable(s)**					
Individual spontaneous yawn frequency	1.542	1.422	1.177	1	0.278
*N*_observations_ = 207, *N*_subjects_ = 86. Random factors: Subject ID, Variance = 0.124, SD = 0.352.					

*Note:* Significant *p* values are in bold; df = degree(s) of freedom; − = not applicable. Estimate ±SE refers to the difference of the response between the reported level of this categorical predictor and the reference category of the same predictor.

**Yawning during affiliative and neutral context in geladas: morphology use according to sex and context, without vocalized yawns (Model 5a); with vocalized yawns (Model 5b)—** ***Prediction 3*** **supported. Model 5a.** Number of observations = 207 unique combinations of context, sex, yawn type for each yawner. Random factor: *Yawner ID* = 86. The full model significantly differed from the null one (*χ* ^2^_11_ = 104.52, *p* < 0.0001). The number of yawns produced by geladas (normalized for context occurrence) was significantly affected by *Context, Yawn type*, *Sex* and by Context*Type, Context*Sex, Yawn type*Sex (Table 2b). Males yawned more during neutral contexts than during affiliation (*β* = −0.937, *p* = 0.002), whereas no significant difference was found for females. During non‐social neutral contexts yawn type 3 were more common compared to yawn type 2 or 1 (type 2 vs. type 3, *β* = −1.179, *p* < 0.0001; type 1 vs. type 3, *β* = −0.720, *p* < 0.0001, Figure 3c), whereas no difference was found during affiliation (for all comparisons, *p* > 0.05); type 3 yawns were less common in the affiliative context (*β* = −0.940, *p* < 0.0001). Finally, males produced more type 3 yawns compared to females and compared to the other morphologies, similarly to previous models' outputs. **Model 5b.** Number of observations = 220 unique combinations of context, sex, yawn type for each yawner also including vocalized yawns. Random factor: *Yawner ID* = 86. The full model differed from the null one (*χ* ^2^_11_ = 106.19, *p* < 0.0001). Similarly to Model 5a, the yawn production (normalized for context occurrence) was significantly affected by *Context, Yawn type*, *Sex* and by Context*Type, Context*Sex, Yawn type*Sex (Table S3b).

### 5.3 Contagious Yawning

**Figure 4:**
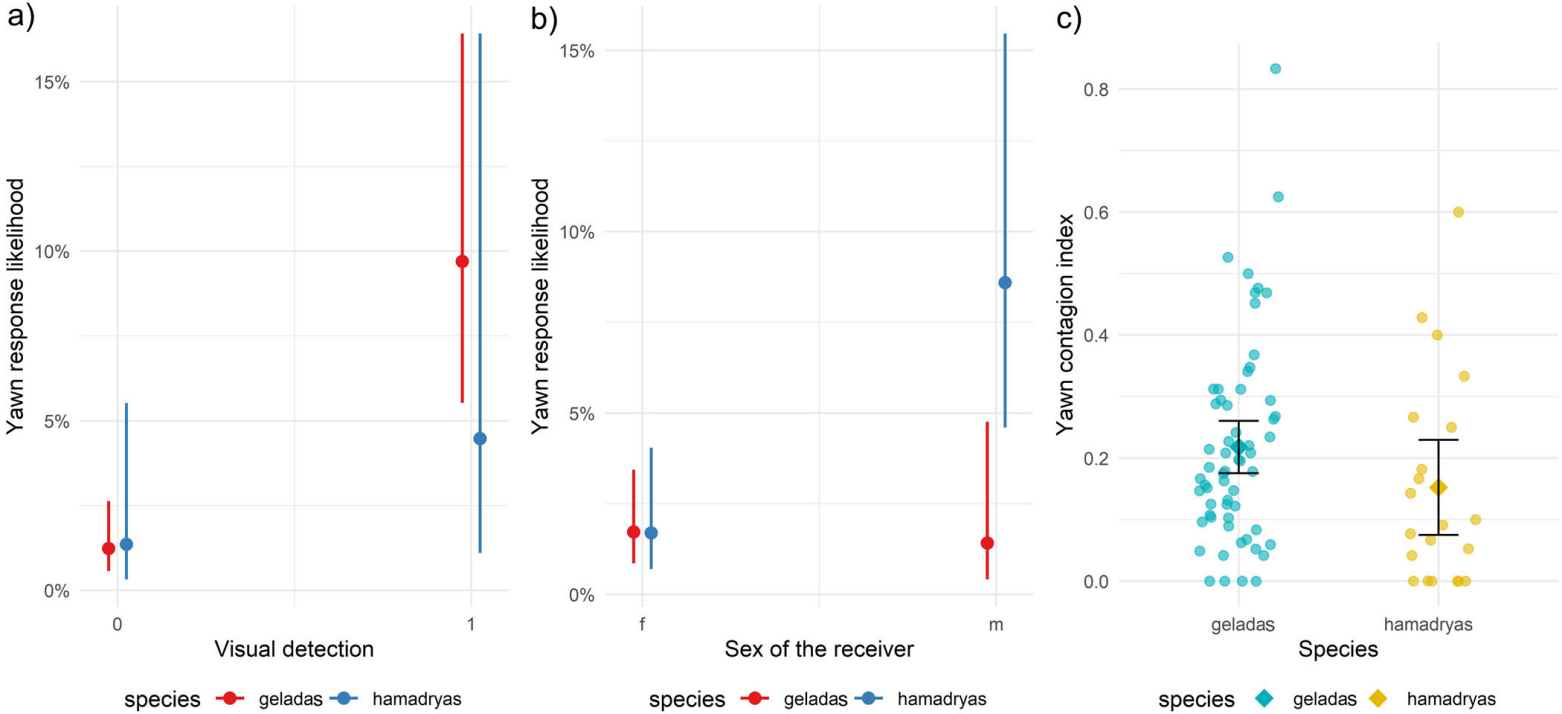
Effect plots showing (a) the significant interaction Visual detection*Species on the likelihood of yawn response in Model 6 (red line = geladas; blue line = hamadryas baboons), (b) the significant interaction Receiver sex*Species on the likelihood of yawn response in Model 6 (red line = geladas; blue line = hamadryas baboons), and (c) the significant effect of the *Species* on the Yawn contagion index in Model 7a (blue = gelada, yellow = hamadryas baboon; each plotted dot represents a subject); error bars represent confidence intervals.

**Model 6. Responding to others' yawns: yawn contagion in the two groups—*Prediction 7, 8, 9*.** Number of observations = 2972, with all vocalized trigger yawns and cases with unclear yawn type excluded. Random factor: Trigger*Receiver combinations = 1043. The full model differed from the control one (*χ* ^2^_12_ = 147.3, *p* < 0.0001). The variables Visual detection*Species (*χ* ^2^ = 6.045, *p* = 0.014, Table 3a) and Receiver sex*Species (*χ* ^2^ = 8.839, *p* = 0.003, Table 3a) significantly affected the likelihood of yawning. Specifically, the likelihood of yawn response was higher after seeing the trigger yawn compared to the control/not seen condition for both groups (Tukey test results, Not seen vs. Seen: geladas, *β* = −2.150, *p* < 0.0001; hamadryas, *β* = −1.227, *p* = 0.0001, Figure 4a, Table 3a) (*Prediction 7* supported). Despite some overlapping in the confidence intervals of the odds ratios, the interaction term between species and perception was statistically significant. The likelihood of yawn response increased by more than 8 times for geladas in the *Seen* condition (average 8.58, range: 5.07–14.52) compared to the control, whereas for hamadryas baboons, the increase was about 3 times (average 3.41, range: 0.97–12.04) (*Prediction 8* supported). Hamadryas males responded more compared to gelada males (*β* = −2.712, *p* = 0.034, Figure 4b) and more than hamadryas females (*β* = −1.692, *p* = 0.0003, Figure 4b) independently from the trigger subject sex (*Prediction 9* supported). On the other hand, gelada males and females and females of the two species did not differ in their likelihood of responding to others' yawns. *Trigger sex, Type of trigger yawn, Duration of trigger yawn*, and *Grooming index* did not affect the response variable (Table 3a).

**Table 3:** Estimated parameters (Coeff), Standard Error (SE), and results of the Likelihood Ratio Tests (*χ* ^2^) of the GLMMs.

Fixed Effects	Coeff	SE	*χ* ^2^	df	*p*
a. *Model 6. Yawn contagion in the two groups. Prediction 7, 8, 9.*					
Intercept	−4.141	0.550	—	—	—
**Tested variables**					
Visual detection (Yes)	2.150	0.268	71.331	1	**0.000**
Trigger sex (Male)	−0.539	0.403	0.770	1	0.380
Receiver sex (Male)	−0.633	0.572	16.498	1	**0.000**
Species (Hamadryas)	0.626	0.866	0.595	1	0.595
Grooming index	−0.502	1.267	0.198	1	0.656
Proximity index	−0.714	1.910	0.006	1	0.938
Type of trigger yawn	—	—	0.740	2	0.740
Type 2	−0.246	0.393	—	—	—
Type 3	0.069	0.233	—	—	—
Duration of trigger yawn	−0.038	0.050	0.560	1	0.560
Visual detection*Species	−0.923	0.375	6.045	1	**0.014**
Trigger sex*Receiver sex	0.838	0.789	0.933	1	0.334
Trigger sex*Species	0.285	0.806	0.000	1	0.997
Receiver sex*Species	2.207	0.885	8.839	1	**0.003**
Trigger sex*Receiver sex*Species	−0.602	1.151	0.274	1	0.274
Grooming index*Species	0.293	1.463	0.040	1	0.841
Proximity index*Species	1.358	2.867	0.224	1	0.636
**Control variables**					
Individual spontaneous yawn frequency	15.475	3.764	16.900	1	0.000
Audience size	−0.075	0.040	9.911	1	0.002
Audience size*Species	−0.109	0.073	2.195	1	0.138
*N*_observations_ = 2972, *N*_trigger‐receiver dyads_ = 1043, *N*_trigger yawns_ = 768. Random factors: Trigger:Receiver, Variance = 0.75, SD = 0.87; Trigger yawn ID, Variance = 0.65, SD = 0.80.					
*b. Model 7a. Yawn contagion index (vocalized yawns excluded). Prediction 7, 8, 9.*					
Intercept			—	—	—
**Tested variable**					
Species (Hamadryas)	−1.073	0.394	9.094	1	**0.002**
Sex (Males)	−0.139	0.407	0.004	1	0.950
Species*Sex	0.290	0.591	0.241	1	0.623
**Control variables**					
Number of yawns perceived	0.008	0.011	0.569	1	0.451
Individual spontaneous yawn frequency	−3.248	2.896	1.258	1	0.262
*N*_observations_ = 88					
*c. Model 7b. Yawn contagion index (vocalized yawns included). Prediction 7, 8, 9.*					
Intercept	−1.760	0.296	—	—	—
**Tested variable**					
Species (Hamadryas)	−1.039	0.403	8.736	1	**0.003**
Sex (Males)	−0.107	0.416	0.011	1	0.915
Species*Sex	0.168	0.593	0.080	1	0.777
**Control variables**					
Number of yawns perceived	0.016	0.009	3.504	1	0.061
Individual spontaneous yawn frequency	−4.070	2.944	1.910	1	0.167
*N*_observations_ = 89.					

*Note:* Significant *P* values are in bold; df = degree(s) of freedom; − = not applicable. Estimate ±SE refers to the difference of the response between the reported level of this categorical predictor and the reference category of the same predictor.

**Yawn contagion index according to species and sex: without vocalized yawns (Model 7a); with vocalized yawns (Model 7b)—*Prediction 7, 8, 9*. Model 7a.** Number of observations = 88, with all subjects who perceived less than three yawns excluded. The full model significantly differed from the control one (*χ* ^2^_3_ = 8.64, *p* = 0.034). The *Species* (*χ* ^2^ = 5.67, *p*= 0.017, Table 3b) significantly affected the *Yawn contagion index*, as hamadryas responded on average in less cases to perceived yawns compared to geladas (*Prediction 8* supported, Figure 4c). On the other hand, *Sex* and Sex*Species did not affect the *Yawn contagion index* of the study subjects (*Prediction 7* and *8* not supported, Table 3b). **Model 7b.** Number of observations = 89. The full model differed from the control one (*χ* ^2^_3_ = 8.23, *p* = 0.041). The *Species* (*χ* ^2^ = 5.67, *p* = 0.017, Table 3c) significantly affected the *Yawn contagion index*, as, similarly as for Model 7a, hamadryas baboons showed lower indication of yawn contagion compared to geladas.

## 6 Discussion

Here we studied the use and complexity (morphology, sensory modalities, contextual use, contagious effect) of yawning to unveil its possible evolutionary advantages. We focused on two zoo‐housed groups of phylogenetically close species showing a similar social structure and differing in their communicative complexity and sociobiology: geladas (*Theropithecus gelada*) and hamadryas baboons (*Papio hamadryas*) (Table 4 summarizes the results of our work). Some of our data support inertia in the evolutionary pathway of yawning, whereas other data support a rapid divergence for the trait in these closely related species. Both species produced three morphological variants according to the exposure of teeth and gums, although only geladas had complete upper lip flip during type 3 yawns, in agreement with their derived upper lip mobility (Lazow and Bergman 2020). However, when considering both silent and vocalized yawns, we found that geladas spontaneously yawned more frequently than hamadryas baboons (*Prediction 1* supported). Moreover, only geladas produced distinct vocalizations associated with yawns, confirming the derived nature of yawn sounds (*Prediction 2* supported) (Gustison et al. 2012) and the link between the emergence of multimodality and social complexity (Peckre et al. 2019). When excluding such vocalized yawns, geladas and hamadryas yawned with comparable frequencies. Altogether, these results hint that acoustic cues in yawns might add a first layer of complexity to the behavior. Moreover, even though both species often performed the behavior in neutral non‐social contexts (i.e., inertia), gelada yawns encompassed multiple contexts (*Prediction 3* supported). The group of hamadryas baboons never yawned in the context of affiliation (i.e., before or during being groomed or grooming a conspecific), while this was the most common context of yawn production in geladas, especially for females. Yawning in non‐social contexts was more common in the group of hamadryas than in geladas. This highlights that yawn complexity goes beyond the sensory modality recruited and extends to the nuanced contextual valence of yawn production. The emotional significance of yawning remains largely uncertain (Diana and Kret 2025), as yawns have been considered as signals of neutral or slightly negative emotional valence (Demuru and Palagi 2012; Leone et al. 2014; Paukner and Anderson 2006; Vick and Paukner 2010; Zannella et al. 2021); yet, our data indicates that in geladas yawns can be associated with contexts of positive emotional valence. Going more in detail, different morphological variants might also be associated with a different affective valence, with type 3 yawns often found in threatening or negatively valent contexts (Leone et al. 2014); in partial accordance with this view, here geladas produced less yawns showing gums and teeth in affiliative social contexts. These results should thus be considered to better understand the function of yawning in rapidly diverged taxa with evolving social needs.

**Table 4:** Summary of the main results of the present study.

	Group 1 (*Theropithecus gelada*)	Group 2 (*Papio hamadryas*)	Relation to tested predictions (If applicable; NA)
Yawn morphologies	Type 1, 2, 3 (with lip‐flip)	Type 1, 2, 3 (no lip‐flip)	NA
Yawn vocalizations	Yes	No	*Prediction 2* supported
Frequency of spontaneous yawning	Higher (when including vocalized yawns)	Lower	*Prediction 1* partially supported
Yawn duration	Shorter	Longer	NA
Sexual dimorphism in yawning frequency	Yes (only when including vocalized yawns)	Yes	*Prediction 4* partially supported
Sexual dimorphism in yawn duration	No	No	*Prediction 5* not supported
Sexual dimorphism in yawn type emission	Yes	Yes	*Prediction 6* supported
Yawning in neutral non‐social contexts	Yes	Yes	*Prediction 3* supported
Yawning in affiliative and post‐copulative contexts	Yes	No	*Prediction 3* supported
Yawn contagion (YC)	Yes (more frequent)	Yes (less frequent)	*Prediction 7* supported; *Prediction 8* supported
Sex‐biased YC	No	Yes	*Prediction 9* supported
Effect of dyadic grooming index on YC	No	No	NA
Effect of dyadic proximity index on YC	No	No	NA

Again, hinting at inertia in the evolution of yawning, in both groups males yawned more often than females with more yawns exposing canines and gums, independently from the context of production (*Prediction 4* partially supported, *Prediction 5* not supported, *Prediction 6* supported). However, when considering silent yawns, only hamadryas baboons showed sexual dimorphism in spontaneous yawning (*Prediction 4* partially supported), suggesting that vocalized yawns might make the difference in spontaneous yawning between the two groups and a greater importance of female yawning in geladas (Pedruzzi et al. 2025) compared to other highly sexually dimorphic species.

In both groups seeing others' yawns led to yawn contagion (YC). Thus, we present evidence of YC also in the *Papio* genus (*Prediction 7* supported), expanding the list of monkey species expressing YC, now including hamadryas along with geladas (Palagi et al. 2009), red‐capped mangabeys (Pedruzzi et al. 2022), drills (Galotti et al. 2024), and spider monkeys (Valdivieso‐Cortadella et al. 2023). Since group living requires synchronization and efficient communication, the presence of YC in *Papio* species is not surprising due to baboon complex social dynamics (Fischer et al. 2019). Considering the sole visual component, geladas were more susceptible to others' yawns compared to hamadryas (*Prediction 8* supported). This result is in line with the greater communicative complexity generally shown by geladas compared to *Papio* species (though direct comparisons are rare in the literature, Gustison et al. 2012; Gustison and Bergman 2017). On the one hand, the need for geladas to maintain long term group cohesion in their larger group units (Grueter et al. 2020) might also be reflected in the tendency to be more easily infected by others' yawns; on the other hand, other tactics known to be commonly used by males (e.g., female coercion and herding, aggressiveness) might be more efficient for hamadryas baboon group cohesion (Amann et al. 2017; Pines et al. 2011). Hamadryas males had a greater tendency to respond to others' yawns not only compared to females but also to gelada males, which here showed similar YC as gelada females (*Prediction 9* partially supported). The centrality of male baboons in YC highlights the known sex‐related differences among the two species, with males being responsible for unit cohesion in hamadryas, whereas the absence of male centrality in contagious yawning in the group of geladas is in line with females being relatively more central characters for group stability in the species (Grueter et al. 2020; Palagi et al. 2009; Snyder‐Mackler et al. 2012).

As a whole, sex‐related differences in spontaneous and contagious yawning are stronger in the group of hamadryas compared to that of geladas, with male hamadryas possibly playing relevant roles in the group dynamics (Amann et al. 2017; Swedell et al. 2011). Importantly, since our data is derived from only two groups living in zoos, generalizations at the species level require caution. For instance, when only considering the data on geladas, a recent study on the same group found female‐female YC to be stronger than in the other sex combinations (Pedruzzi et al. 2025), in line with previous data (Palagi et al. 2009); such effect seems however to be reduced here when including also hamadryas yawns in the analyses; moreover, another study seems to hint at a more responsive role of males compared to females in the wild (Gallo et al. 2021, but note that the authors did not control for the spontaneous yawning frequency of the study subjects, which is higher in males and strongly affects the tendency to respond, independently from the occurrence of actual YC). Further research and protocol standardization are thus needed to compare more groups of *Theropithecus gelada* and *Papio hamadryas* from wild populations, to better comprehend the socio‐ecological relevance of differences in spontaneous and contagious yawning or other signaling and communicative strategies.

Although numerous studies have supported the social complexity hypothesis by identifying indirect correlations between social and communicative variables, a direct link between variation in signaling and social factors through direct cross‐species comparisons is not usually explored (Freeberg et al. 2012; Gustison et al. 2012; Manser et al. 2014; Peckre et al. 2019). Geladas appear to have undergone a recent and rapid divergence in communicative traits, as previously demonstrated for their derived vocal repertoire (Gustison et al. 2012; Gustison and Bergman 2017). We propose that this divergence may extend to yawning behavior, with key indicators including its multimodal nature, increased production frequency, broader contextual usage—potentially reflecting more diverse functions—and its heightened contagiousness. These factors suggest that yawning in geladas has evolved beyond its ancestral form in other Papionine species, aligning with their complex social dynamics and communicative needs.

To conclude, our findings suggest that geladas exhibit more nuanced and contextually varied yawning behavior, likely tied to their rich communicative needs and social structures, whereas hamadryas baboons show a more male‐centric pattern of yawning, reflecting their distinct social dynamics. These results prompt new research on yawn contagion, especially in baboons and wild populations, to fully understand the possible meaning of yawns and yawn contagion in primates living in complex social systems. Future research will need to investigate the affective value associated with yawn vocalizations to better understand the role of the acoustic component in increasing yawn complexity.

## Author Contributions

**Luca Pedruzzi:** conceptualization (equal), data curation (equal), formal analysis (lead), investigation (equal), methodology (equal), visualization (lead), writing – original draft (lead), writing – review and editing (equal). **Veronica Maglieri:** conceptualization (equal), data curation (equal), investigation (equal), methodology (equal), writing – review and editing (equal). **Paolo Oliveri:** data curation (equal), methodology (equal). **Martina Francesconi:** data curation (equal), methodology (equal), visualization (equal). **Rea Riccobono:** data curation (equal), methodology (equal). **Filippo Bigozzi:** data curation (equal), methodology (equal). **Alban Lemasson:** conceptualization (equal), investigation (equal), project administration (equal), supervision (equal), writing – review and editing (equal). **Elisabetta Palagi:** conceptualization (equal), investigation (equal), project administration (equal), supervision (equal), writing – review and editing (lead).

## Conflicts of Interest

The authors declare no conflicts of interest.

## Supporting information

Table S1.

Table S2 R1.

Table S3 R1.

Raw data.

## Data Availability

All data used for the present study have been uploaded as supporting materials.
