# Supplementary material for: Inertia and Rapid Divergence in the Evolution of Yawning: A Comparison Between Two Closely Related but Socially Different Monkeys
Source: Am J Primatol. 2025 May 29;87(6):e70049. doi: 10.1002/ajp.70049 (PMC12120385; doi:10.1002/ajp.70049)
Supplement: Supplementary file 1 — Table S1. [file AJP-87-e70049-s002.docx]

**Table S1. a)** Individually recognized geladas hosted at NaturZoo Rheine (Germany) in 2023 and **b)** hamadryas baboons hosted at Hellabrunn Munich Zoo (Germany) in 2019.

**a)**

| **SUBJECT** | **ENCLOSURE** | **SEX** |
| --- | --- | --- |
| Biondo | G1 | M |
| Barbie | G1 | F |
| Basic | G1 | F |
| Belly | G1 | F |
| Bernoccolo | G1 | M |
| Betta | G1 | F |
| Biba | G1 | F |
| Bifida | G1 | F |
| Bigne | G1 | F |
| Bijoux | G1 | F |
| Bisnonna | G1 | F |
| Black | G1 | M |
| Blanca | G1 | F |
| Blind | G1 | F |
| Borsa | G1 | F |
| Bortolo | G1 | M |
| Braccio | G1 | F |
| Bratz | G1 | F |
| Giangi | G1 | M |
| Gatta | G1 | F |
| Gelly | G1 | F |
| Gessica | G1 | F |
| Ghiozza | G1 | F |
| Ghirlanda | G1 | F |
| Gianna | G1 | F |
| Gigetto | G1 | M |
| Gigio | G1 | M |
| Grappolo | G1 | F |
| Enzo | G2 | M |
| E1m6 | G2 | F |
| E2m6 | G2 | F |
| Edera | G2 | F |
| Elly | G2 | F |
| Evasa | G2 | F |
| Strip | G2 | F |
| Striscia | G2 | F |
| Sumo | G2 | M |
| Neomamma | G2 | F |
| Osso | G2 | F |
| Roll | G2 | F |
| Rosa | G2 | F |
| Ruga | G2 | F |
| Sally | G2 | F |
| Scapola | G2 | F |
| Schiarita | G2 | F |
| Sciura | G2 | F |
| Secco | G2 | M |
| Severa | G2 | F |
| Sfasciato | G2 | M |
| Sguercia | G2 | F |
| Sine | G2 | F |
| Small | G2 | F |
| Sonia | G2 | F |
| Sorcia | G2 | F |
| Spiga | G2 | F |
| Spilla | G2 | F |
| Spot | G2 | F |
| Stacy | G2 | F |
| Stella | G2 | F |
| Strega | G2 | F |
| Susy | G2 | F |
| Tris | G2 | F |
| Rocco | G2 | M |
| Sandro | G2 | M |
| Tino | G2 | M |
| Tinino 1 | G2 | M |
| Tinino 2 | G2 | M |

**b)**

| SUBJECT | SEX |
| --- | --- |
| Buco prossimale | M |
| Buco Sx | F |
| Catta | M |
| Cicciona | F |
| Cometa Sx | M |
| H | F |
| Indiano | M |
| Naso | M |
| Orango | M |
| Orecchio Tagiato | F |
| Ovale | F |
| Palpebra | F |
| Pandorina | F |
| Patacorta | F |
| Perfettina | F |
| Perfetto | M |
| Perpendicolare | M |
| Pisellina | F |
| Polso | M |
| Puntino | M |
| Rossonero | M |
| Sfregiato | M |
| Spallasfatta | F |
| Sterna | F |
| Tre | F |
| V | F |
| Vecchia | F |
| Verruca | M |
