# Supplementary material for: Inertia and Rapid Divergence in the Evolution of Yawning: A Comparison Between Two Closely Related but Socially Different Monkeys
Source: Am J Primatol. 2025 May 29;87(6):e70049. doi: 10.1002/ajp.70049 (PMC12120385; doi:10.1002/ajp.70049)
Supplement: Supplementary file 2 — Table S2 R1. [file AJP-87-e70049-s003.docx]

**Table S2.** Estimated parameters (Coeff), Standard Error (SE), and results of the Likelihood Ratio Tests (**χ^2^**) of the GLMMs. Significant *P* values are in bold; df= degree(s) of freedom; - = not applicable. Estimate ± SE refers to the difference of the response between the reported level of this categorical predictor and the reference category of the same predictor.

| **Fixed Effects** | **Coeff** | **SE** | **χ^2^** | **df** | ***P*** |  |
| --- | --- | --- | --- | --- | --- | --- |
| 1. ***Model 2b - Yawn duration (vocalized yawns included) - Prediction 5*** | | | | | |  |
| Intercept | 0.629 | 0.028 | - | - | - |  |
| **Tested variables** | | | | | |  |
| Sex | -0.106 | 0.053 | 1.951 | 1 | 0.163 |  |
| Species | 0.215 | 0.081 | 119.399 | 1 | **0.000** |  |
| Type | - | - | 112.273 | 2 | **0.000** |  |
| Type 2 | 0.004 | 0.100 | - | - | - |  |
| Type 3 | 0.146 | 0.037 | - | - | - |  |
| Sex*Species | 0.145 | 0.108 | 0.931 | 1 | 0.335 |  |
| Sex*Type | - | - | 0.390 | 2 | 0.822 |  |
| Male:Type 2 | 0.080 | 0.109 | - | - | - |  |
| Male:Type 3 | 0.045 | 0.050 | - | - | - |  |
| Species*Type | - | - | 17.931 | 2 | **0.000** |  |
| Hamadryas:Type 2 | 0.260 | 0.144 | - | - | - |  |
| Hamadryas:Type 3 | 0.275 | 0.089 | - | - | - |  |
| Sex*Species*Type | - | - | 1.043 | 2 | 0.594 |  |
| Male:Hamadryas:Type 2 | -0.147 | 0.167 | - | - | - |  |
| Male:Hamadryas:Type 3 | -0.092 | 0.108 | - | - | - |  |
| N_observations_=1779, N_subjects_=102. Random factors: Subject ID, Variance=0.016, SD=0.126. | | | | | |  |
| 1. ***Model 3b - Yawn morphology (vocalized yawns included) - Prediction 6*** | | | | | | |
| Intercept | | 1.320 | 0.126 | - | - | - |
| **Tested variables** | | | | | | |
| Sex | |  |  | 5.779 | 1 | **0.016** |
| Species | | -0.824 | 0.338 | 1.766 | 1 | 0.184 |
| Type | | - | - | 60.201 | 2 | **0.000** |
| Type 2 | | -1.431 | 0.362 | - | - | - |
| Type 3 | | -0.417 | 0.150 | - | - | - |
| Sex*Species | | - | - | 13.621 | 1 | **0.000** |
| Sex*Type | | - | - | 15.631 | 2 | **0.000** |
| Male:Type 2 | | 0.707 | 0.459 | - | - | - |
| Male:Type 3 | | 0.931 | 0.282 | - | - | - |
| Species*Type | | - | - | 13.235 | 2 | **0.001** |
| Hamadryas:Type 2 | | 1.239 | 0.582 | - | - | - |
| Hamadryas:Type 3 | | 0.944 | 0.379 | - | - | - |
| Sex*Species*Type | | - | - | 0.991 | 2 | 0.609 |
| Male:Hamadryas:Type 2 | | -0.679 | 0.696 | - | - | - |
| Male:Hamadryas:Type 3 | | -0.142 | 0.491 | - | - | - |
| **Control variable(s)** | | | | | | |
| Individual spontaneous yawn frequency | | 10.337 | 1.794 | 33.205 | 1 | 0.000 |
| N_observations_=167, N_subjects_=86. Random factors: Subject ID, Variance=0.174, SD=0.417. | | | | | | |
