# Supplementary material for: Inertia and Rapid Divergence in the Evolution of Yawning: A Comparison Between Two Closely Related but Socially Different Monkeys
Source: Am J Primatol. 2025 May 29;87(6):e70049. doi: 10.1002/ajp.70049 (PMC12120385; doi:10.1002/ajp.70049)
Supplement: Supplementary file 3 — Table S3 R1. [file AJP-87-e70049-s004.docx]

**Table S3.** Estimated parameters (Coeff), Standard Error (SE), and results of the Likelihood Ratio Tests (**χ^2^**) of the GLMMs. Significant *P* values are in bold; df= degree(s) of freedom; - = not applicable. Estimate ± SE refers to the difference of the response between the reported level of this categorical predictor and the reference category of the same predictor.

| **Fixed Effects** | **Coeff** | **SE** | **χ^2^** | **df** | ***P*** |  |
| --- | --- | --- | --- | --- | --- | --- |
| 1. **Model 4b. Yawning in neutral context (vocalized yawns included).** | | | | | | |
| Intercept | | 0.884 | 0.143 | - | - | - |
| **Tested variables** | | | | | | |
| Sex (Male) | | -0.588 | 0.284 | 15.496 | 1 | **0.000** |
| Species (Hamadryas) | | -0.356 | 0.326 | 4.428 | 1 | **0.035** |
| Type | | - | - | 90.068 | 2 | **0.000** |
| Type 2 | | -1.158 | 0.498 | - | - | - |
| Type 3 | | 0.069 | 0.175 | - | - | - |
| Sex*Species | | 1.228 | 0.436 | 15.194 | 1 | **0.000** |
| Sex*Type | | - | - | 14.577 | 2 | **0.001** |
| Male:Type 2 | | 0.371 | 0.619 | - | - | - |
| Male:Type 3 | | 0.849 | 0.298 | - | - | - |
| Species*Type | | - | - | 5.104 | 2 | 0.078 |
| Hamadryas:Type 2 | | 0.970 | 0.662 | - | - | - |
| Hamadryas:Type 3 | | 0.449 | 0.374 | - | - | - |
| Sex*Species*Type | | - | - | 0.195 | 2 | 0.907 |
| Male:Hamadryas:Type 2 | | -0.337 | 0.796 | - | - | - |
| Male:Hamadryas:Type 3 | | -0.029 | 0.482 | - | - | - |
| **Control variable(s)** | | | | | | |
| Individual spontaneous yawn frequency | | 7.957 | 1.726 | 21.239 | 1 | 0.000 |
| N_observations_=146, N_subjects_=76. Random factors: Subject ID, Variance=0.103, SD=0.320. | | | | | | |
| 1. **Model 5b. Yawning during affiliative and neutral context in geladas (vocalized yawns included)***.* | | | | | | |
| Intercept | | 1.205 | 0.137 | - | - | - |
| **Tested variables** | | | | | | |
| Sex (Male) | | -0.676 | 0.232 | 12.679 | 2 | **0.000** |
| Context (Non-social neutral) | | -0.332 | 0.169 | 3.049 | 1 | **0.000** |
| Type | | - | - | 48.514 | 2 | **0.000** |
| Type 2 | | -0.722 | 0.507 | - | - | - |
| Type 3 | | -0.676 | 0.232 | - | - | - |
| Context*Sex | | 0.622 | 0.306 | 7.89 | 1 | **0.017** |
| Sex*Type | | - | - | 31.547 | 2 | **0.000** |
| Male:Type 2 | | 0.824 | 0.619 | - | - | - |
| Male:Type 3 | | 1.064 | 0.379 | - | - | - |
| Context*Type | | - | - | 20.510 | 2 | **0.004** |
| Non-social neutral:Type 2 | | -0.044 | 0.594 | - | - | - |
| Non-social neutral:Type 3 | | 0.815 | 0.284 | - | - | - |
| Context*Sex*Type | | - | - | 0.324 | 2 | 0.850 |
| Male: Non-social neutral:Type 2 | | -0.333  3 | 0.725 | - | - | - |
| Male: Non-social neutral:Type 3 | | 0.087 | 0.444 | - | - | - |
| **Control variable(s)** | | | | | | |
| Individual spontaneous yawn frequency | | 4.527 | 1.320 | 11.756 | 1 | 0.001 |
| N_observations_=220, N_subjects_=86. Random factors: Subject ID, Variance=0.143, SD=0.378. | | | | | | |
